# Alteration of Daily and Circadian Rhythms following Dopamine Depletion in MPTP Treated Non-Human Primates

**DOI:** 10.1371/journal.pone.0086240

**Published:** 2014-01-23

**Authors:** Karim Fifel, Julien Vezoli, Kwamivi Dzahini, Bruno Claustrat, Vincent Leviel, Henry Kennedy, Emmanuel Procyk, Ouria Dkhissi-Benyahya, Claude Gronfier, Howard M. Cooper

**Affiliations:** 1 Inserm, U846, Stem Cell and Brain Research Institute, Bron, France; 2 Université de Lyon, Lyon 1, UMR-S 846, Lyon, France; Simon Fraser University, Canada

## Abstract

Disturbances of the daily sleep/wake cycle are common non-motor symptoms of Parkinson's disease (PD). However, the impact of dopamine (DA) depletion on circadian rhythms in PD patients or non-human primate (NHP) models of the disorder have not been investigated. We evaluated alterations of circadian rhythms in NHP following MPTP lesion of the dopaminergic nigro-striatal system. DA degeneration was assessed by *in vivo* PET ([^11^C]-PE2I) and post-mortem TH and DAT quantification. In a light∶dark cycle, control and MPTP-treated NHP both exhibit rest-wake locomotor rhythms, although DA-depleted NHP show reduced amplitude, decreased stability and increased fragmentation. In all animals, 6-sulphatoxymelatonin peaks at night and cortisol in early morning. When the circadian system is challenged by exposure to constant light, controls retain locomotor rest-wake and hormonal rhythms that free-run with stable phase relationships whereas in the DA-depleted NHP, locomotor rhythms are severely disturbed or completely abolished. The amplitude and phase relations of hormonal rhythms nevertheless remain unaltered. Use of a light-dark masking paradigm shows that expression of daily rest-wake activity in MPTP monkeys requires the stimulatory and inhibitory effects of light and darkness. These results suggest that following DA lesion, the central clock in the SCN remains intact but, in the absence of environmental timing cues, is unable to drive downstream rhythmic processes of striatal clock gene and dopaminergic functions that control locomotor output. These findings suggest that the circadian component of the sleep-wake disturbances in PD is more profoundly affected than previously assumed.

## Introduction

Disturbances of the sleep-wake timing are a prominent feature of non-motor symptoms of Parkinson's disease (PD), expressed as insomnia, excessive daytime sleepiness and fragmentation of sleep architecture [1], [2]. While the loss of dopaminergic neurons in the nigro-striatal pathway is the main neuropathological feature and cause of motor disabilities in PD [3], dopamine (DA) is not generally considered to play a significant role in sleep or circadian rhythms and other neurotransmitters such as noradrenaline, serotonin and orexin have been proposed [4]. However, DA is known to be an important wake promoting factor across species [5], [6] and has recently been shown to be closely linked with circadian clock function. Almost all aspects of dopaminergic transmission exhibit daily rhythms of expression [7]–[9] that are both light and clock dependent [10], [11]. Inversely, DA has been shown to modulate clock gene expression both *in vivo* and *in vitro* [8], [12], [13] and lesion of the nigro-striatal system disrupts the daily and circadian rhythms of rest-activity and striatal clock gene expression [13].

Given the association between DA and circadian processes and the fundamental role of the circadian system in the determination of sleep timing [14], [15] we explored the alterations of daily and circadian rest-wake activity and hormonal rhythms in the DA depleted MPTP-treated non-human primate (NHP). The MPTP-treated NHP is considered a highly appropriate model of PD since, in addition to cognitive and motor deficits, it closely replicates several features of sleep and wake abnormalities, including excessive daytime sleepiness, REM sleep disturbances and altered day-night activity patterns. Many of these precede the appearance of motor symptoms and have been proposed as early biomarkers of the premotor phase of PD [1], [16], [17]. In contrast, other output rhythms controlled by the endogenous circadian clock, such as the daily hormonal secretion profiles of melatonin and cortisol do not appear to be significantly altered in either MPTP monkeys [18] or in PD patients [19], [20], [21]. A critical limitation of all these studies is that investigations were conducted under a light/dark cycle that exerts strong acute and chronic effects on daily physiology and behaviors [22], [23]. Because the excitatory and inhibitory influences of light and dark can potentially over-ride circadian clock control, we examined the rest/activity rhythms of MPTP-treated NHP monkey in lighting conditions devoid of any cyclic environmental timing cues to determine the impact of DA neurodegeneration on circadian functions.

Our findings show that in constant conditions, rhythmic rest-wake activity following DA depletion is strongly attenuated or abolished whereas hormonal rhythms are conserved. In an LD cycle, an altered but coherent 24 h activity rhythm is expressed that can be directly attributed to the excitatory and inhibitory effects of light and darkness. These results argue that the circadian clock remains intact but, in the absence of environmental light-dark cues is unable to drive rhythmic locomotor behaviors, suggesting that the circadian component of the sleep-wake disturbances observed in PD is more profoundly affected than previously assumed. These findings further emphasize that appropriate light exposure is essential for optimal circadian function in PD patients.

## Materials and Methods

### Ethics Statement

All procedures were carried out according to the 1986 European Community Council Directives (86/609/EEC), the French Ministe're de l'Agriculture et de la Forêt, French Commission of animal experimentation, the Department of Veterinary Services (DDSV Lyon, France). These experiments were also carried out according to guidelines published in the Guide for the Care and Use of Laboratory Animals of the National Institutes of Health. Under local rules, authorization is granted to the laboratory for a range of experiments, rather than for specific studies. Specific authorization covering this study was delivered by the “Préfet de la Région Rhône Alpes” and the “Directeur départemental de la protection des populations” under Permit Number: #A690290402, including approved protocols in NHPs (#047, #048, #0198, #0199, #0200). All procedures complied with guidelines for animal welfare in accordance with the recommendations of the Weatherall report, “The use of non-human primates in research”. The nature of the measures and interventions described below required individual housing of monkeys during the experiment. This is also a welfare measure – symptomatic MPTP-treated monkeys would be at risk of persecution in a group-housing situation. Given this, however, all efforts were made to provide an enriched (enrichment toys, Special Diet Services, Argenteuil, France) and social environment for the animals in a colony room with other familiar monkeys. All monkeys were closely monitored on a regular basis throughout the day, by several researchers as well as animal care staff, in order to ensure that levels of health and welfare were maintained. This was particularly the case during the MPTP period. As required, adaptations to housing and feeding procedures were made in direct response to individual symptoms in the MPTP phase, for example adaptations of water provision to ensure monkeys were able to drink ad libitum throughout.

### Animals and housing

Five adult rhesus monkeys (*Macaca mulatta*, 1 female and 4 males) aged between 5–11 years old and six adult female cynomolgus monkeys (*Macaca fascicularis*) aged between 11–14 years old were used. The animals were housed in individual cages in light, temperature-controlled (24–26°C) and sound-proofed rooms dedicated to MPTP and circadian experiments. There was free access to water and animals were fed twice a day with pellets (Special Diets Services), supplemented with fruit. Food was supplied randomly twice a day in the light phase of the LD cycle or on the subjective day during constant conditions. Illumination was provided by white fluorescent tubes (Philips, 15 Watts) mounted on the ceiling above the animal's cage.

### MPTP administration

The eleven drug-naïve monkeys were divided into a group of 4 monkeys used as untreated controls and 7 monkeys that were rendered Parkinsonian with 1-methyl-4-phenyl-1, 2, 3, 6-tetrahydropyridine (MPTP; Sigma, St Louis, Mo, USA). MPTP injections were intramuscular using relatively low doses (0.2 mg/Kg). The neurotoxin was delivered initially every 3–4 days, and in some cases, towards the end of treatment, every 1–2 days as previously described [17]. The administration was continued until animals showed a significant clinical score of motor deficits varying from mildly to highly symptomatic states (see definitions below). The total cumulative doses ranged from 2.2–7.8 mg/kg and the duration of treatment ranged from 18 to 90 days. This mode of MPTP treatment has the advantage of producing behavioural symptoms that closely resemble the clinical features of idiopathic PD and the possibility of titrating the degree of lesion by adjusting the frequency of injections resulting in a range of clinical symptoms [24]. In the text as well as in the analyses, control animals refer to the 4 untreated animals and the 7 treated animals prior to MPTP administration, unless otherwise specified.

### Clinical Motor Evaluation

Motor status was monitored 5 days/week, by at least 2 trained observers, during 10–15 min observation periods using a semi-quantitative Parkinsonian Monkey Rating Scale (PMRS) adapted from the Unified Parkinson's Disease Scale (UPDRS) used for patients and compatible with rating scales used for monkeys [25]. We defined three different states of motor symptom severity: a score of 0 to 5 defined the non-symptomatic stage, a score from 5 to 8.5 corresponded to a mild symptomatic state and a score of ≥8.5 to a severe symptomatic state, in accordance with previous criteria [26]. The use of a score >5 to define the threshold for the symptomatic stage was applied as previously described [17], [25]. The subdivision of the symptomatic stage into mildly and highly symptomatic stages was based on the fact that monkeys with a score >8.5 become increasingly bradykinetic, adopting a flexed posture with increased rigidity of the limbs and less accurate movements when for example reaching for food and with occasional episodes of freezing.

### Assessment of behavioral rhythms

Locomotor activity was continuously recorded in 1 min intervals throughout the entire duration of the study using passive-infrared motion detectors mounted above each animal's cage. The motion captors were connected to a computerized data acquisition system (Circadian Activity Monitoring, INSERM, France; for details see [27]). Data were analysed using the Clocklab software package (Actimetrics, Evanston, IL, USA).

We used Chi-squared periodogram method to calculate the period of behavioral rhythm which is defined as the time elapsed for one complete oscillation or cycle (the distance in time between two consecutive peaks or troughs of a recurring rhythm). The stability and the consistency of the rhythm are reflected by the value of the periodogram amplitude which corresponds to the zenith of a periodogram curve. The subjective day and the subjective night are defined as the segment of a circadian cycle during the free-running state that corresponds to, respectively, the light and dark segments during entrainment by a LD cycle. Finally, activity onset, which is a robust indicator of entrainment to LD cycle, corresponds to the average clock (or circadian) time of activity onset while activity offset to the average clock (or circadian) time of activity offsets. Phase angle is the difference between the time of the activity light onset or offset compared to lights on-lights off.

Non-parametric circadian rhythm analyses (NPCRA) were used to estimate the strength and fragmentation of rest-activity rhythms [28]. These included measurements of the inter-daily stability (calculated as the ratio between the variance of the average 24-hour pattern around the mean and the overall variance and gives an indication of the strength of coupling between the rest-activity rhythm and LD cycle) and the intra-daily variability (calculated as the ratio of the mean squares of the difference between successive hours and the mean squares around the grand mean and gives an indication of the frequency of transitions between rest and activity periods, corresponding to the fragmentation of the rhythm). When the rest-activity rhythm is stable, inter-daily stability is high and the intra-daily variability is low.

### Lighting protocols

Animals were recorded for periods of 8 months to more than 24 months. Prior and after MPTP administration, the animals were maintained under an LD cycle (12 h light∶12 h dark) of approximately 450–500 lux during the light phase and 0 lux during the dark phase. The animals were periodically exposed for 12–15 days to conditions of constant light (LL) of 300 lux. Unlike rodents, diurnal primates do not tolerate constant darkness (DD) that induces stress and is considered unethical practice. On a few occasions continuous dim light (5–10 lux) was examined, but as no differences were found between the two lighting conditions, data was grouped together for analysis. The average time between the end of MPTP treatment and the first exposure to LL was 30–210 days (avg = 96 days) and for subsequent exposures was 60–367 days thereafter. Between two LL periods, the animals were always re-entrained to an LD cycle for at least 4 weeks. Behavioral masking responses were investigated using a 1 h light/1 h dark lighting regime for 4–5 days.

### Functional [11C]-PE2I Binding Potential Evaluation

To evaluate the *in vivo* DA function, images from PET scans using [^11^C]-PE2I (N-(3-iodoprop-2 E-enyl)-2beta-carbomethoxy-3beta-(4-methylphenyl) nortropane) were obtained at different periods throughout the entire protocol. PE2I specifically binds with high affinity and selectivity to DAT (Ki = 17 nM) and is considered to provide an index of the integrity of the DA pathway that has been used for PD diagnosis [29]–[31]. PET studies were performed with an ECAT Exact HR+ tomograph (Siemens CTI), in 3D acquisition mode, covering an axial distance of 15.2 cm. The transaxial resolution of the reconstructed images was about 4.1 mm full-width and half maximum in the center. Transmission scans were acquired with three rotating 68Ge sources and were used to correct the emission scans for the attenuation of 511 Kev photonrays through tissue and head support. The specific procedures of the experimental methods employed at the CERMEP imaging facility (Lyon) ([^11^C]-PE2I labeling, subjects PET examinations, PET Evaluation and modelling) have been described in detail elsewhere [32].

### Hormone radioimmunoassays (RIA)

Nycthymeral and circadian profile of aMT6s and cortisol excretion were studied in control and MPTP-treated animals in both LD and constant conditions. Previous reports have shown that aMT6s assays correlate significantly with daily plasma melatonin profiles and can be used as non-invasive methods to study melatonin secretion [33]–[36]. Urine samples were collected at 3 h intervals for 48 h. In the end of collection, urine samples were filtered, aliquoted and stored at −20°c until assayed. Urinary aMT6s and cortisol levels were determined by radioimmunoassay as previously reported [35]. Quantity rates (quantities/time span) were calculated for each parameter. This resulting expression takes into account a possible dilution of urine related to the hydration of animals. Of the 27 series of assays, 9 were excluded due to an insufficient number of samples during the 48 h sampling period. Day-night concentration ratios were determined for the remaining 18 assays, and 13 of these had a sufficient number of successive samples over a 24 h window within the 48 h sampling period for the dual harmonic regression analysis.

### Brain Immunohistochemistry

Three MPTP treated animals and two controls were euthanized at the end of the protocols to assess brain and retinal cellular markers (see below). The four remaining MPTP animals were maintained for further studies in other protocols. The remaining control animals were transferred to an approved semi-natural retirement facility. Anesthesia was induced with Ketamine after premedication with chlorpromazine hydrochloride (Largactil™) followed by a lethal dose of pentobarbital sodium (60 mg.kg-1, i.p.) before animals were transcardially perfused with saline (0.9% with procaine) and 4% paraformaldéhyde and 0.05%-glutaraldehyde in phosphate buffer (PB). Cryoprotection was ensured by sucrose gradients (10–30%) perfusion post fixation. Brains were removed, kept in cryoprotecting liquid overnight and coronal 50 µm thick sections were cut on a freezing microtome. Free floating sections were rinsed briefly in PB (0.01 M, pH 7.4) containing 0.9% saline, 0.3% triton X-100 and 0.1% sodium azide (PBSTA) and blocked with 1.5% normal serum. Sections were then incubated with antibodies against Orexin A (1∶50.000, rabbit, Phoenix Pharma #H-003-30) tyrosine hydroxylase (TH; 1∶1000, mouse, Millipore #MAB318), DAT (Millipore, #MAB369), or vasoactive intestinal peptide (VIP; 1∶10.000, rabbit, a generous gift of Prof. Jen Mikkelsen) at 4°C for 72 h. After rinsing twice in PBSTA, sections were incubated with biotinylated secondary antibodies for 2 h (1∶200, Vector) and in an avidin-biotin-horseradish peroxidase complex (1∶200, Sigma-Aldrich) at 22–24°c. The reaction product was visualized with 3,3′-diaminobenzidine as the chromogen with 0.5% ammonium nickel sulphate and 0.03% H_2_O_2_ in TRIS buffer. Sections received additional rinses, were mounted on gelatinized slides, dried and dehydrated in increasing gradients of ethanol, cleared in toluene and were coverslipped with Depex.

#### Semi-quantitative immunohistochemistry

Evaluation of the extent and amplitude of the lesion in the SN-VTA complex and the striatal structures was obtained with semi-quantitative immunohistochemistry [37]. The mean optical density (O.D.) of TH and DAT immunoreactive regions was computed on at least 5 sections (distance between sections 150 µm) per animal (range; 5–20) from 8bit images. All hypocretin-1 expressing neurons in the hypothalamus were counted on 5–7 sections per animal (distance between sections 150–200 µm).

### Retinal Immunohistochemistry

Following the perfusions, the retina was gently dissected from the eyecup, and post-fixed for 24 h in 4% buffered paraformaldehyde. To digest the vitreous and enhance penetration, the retinas were pre-treated with a mixture of collagenase-hyaluronidase in TBS/CaCl_2_ (10 mM) at 37°c for 30 min, and then rinsed with TRIS buffer (0.05M). Endogenous peroxidase activity was suppressed by treatment with a solution of 50% ethanol in saline with 0.4% H_2_O_2_ for 1 h. After a blocking step (1.5% normal serum, 0.3% triton X-100, 0.1% sodium azide in saline, 1 h, 4°C), tissue was incubated for 3 days in primary antibodies against TH (1∶1000, Millipore, #MAB369) or Melanopsin (1/4000, a generous gift of Prof. I. Provencio, USA) at 4°C. After two PBST washes (10 min, 20°C), flat mounts were incubated for 2 hours with biotinylated secondary antibodies diluted 1∶200. Thereafter, the avidin-biotin-peroxidase reaction (1∶200, Sigma-Aldrich) was carried out using diaminobenzidine as the chromogen. After a final 2 washes in TBS (10 min, 4°C) the flat-mounts were mounted in gelatinized slides and were coverslipped with Depex. The density of melanopsin and TH immunopositive neurons was evaluated in the flat mounted retinas by counts of the average number of cells in sampling areas of 0.38 mm^2^ spaced 3 mm apart.

### Analysis and Statistics

The calculation of a significant behavioral rhythm was performed using Chi-squared periodogram method [38]. For analysis of behavioural amplitudes at different clinical stages, cosinor analysis and a Linear mixed model (lme4 package) in R (version 2.12.2) were used. For analysis of hormonal rhythms, the dual harmonic regression model was used to estimate the amplitude and the acrophase of the rhythms (Sigmaplot 12, Systat Software Inc.). Behavioral parameters were analysed using a two-way analysis of variance ANOVA (with treatments and light conditions as factors). *Post hoc* comparisons of interactions were performed with the Bonferroni correction. For analysis of the TH, melanopsin and orexin-A positive cell, a one-way analysis of variance ANOVA was used. P values less than 0.05 were considered statistically significant.

## Results

### MPTP-treated non-human primates show a loss or reduction of circadian rhythmicity under constant conditions

**Figure 1:**
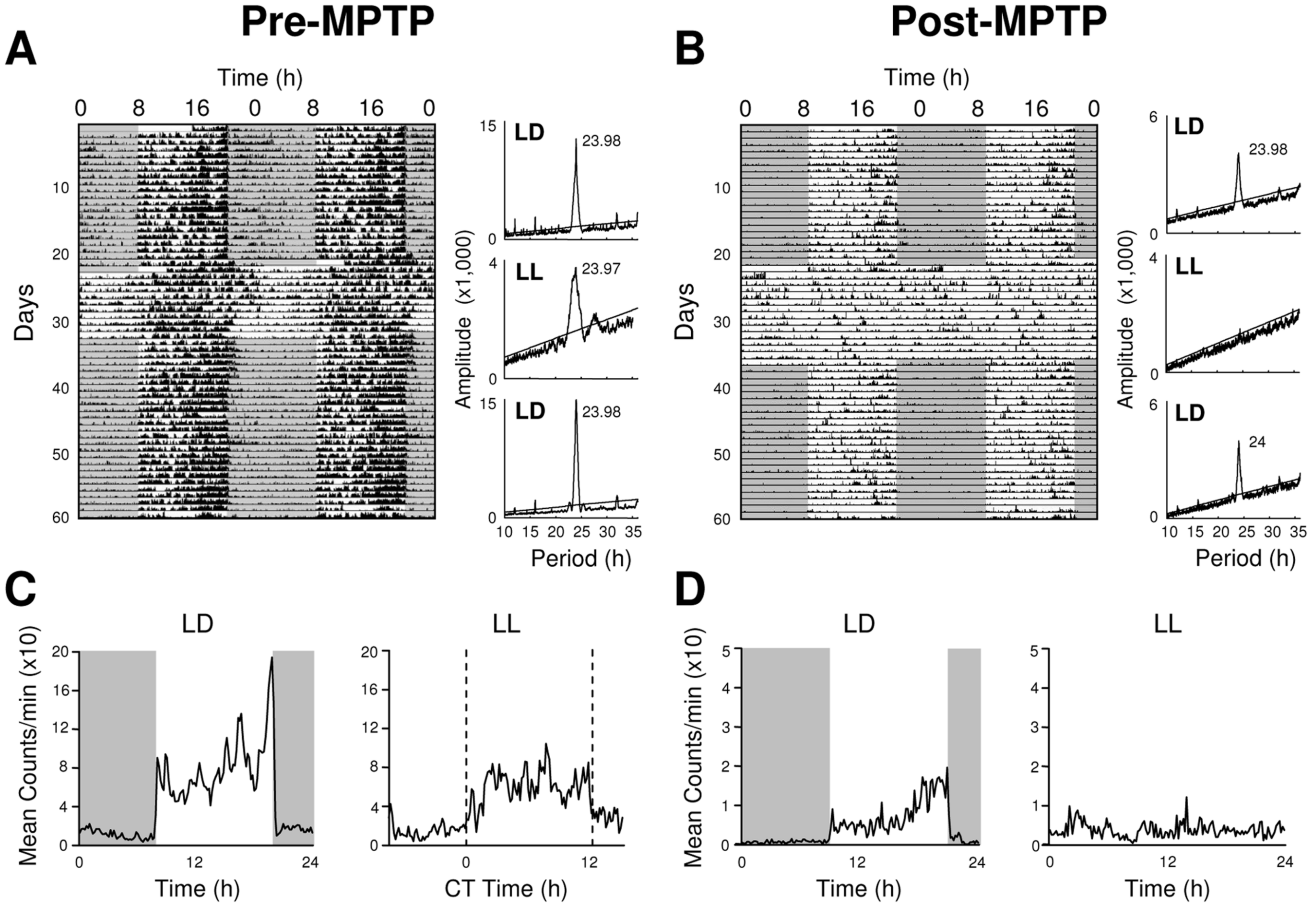
Following MPTP treatment, the circadian locomotor rest-activity rhythm is abolished under constant light. (**A**) Pre-MPTP treatment, daily locomotor activity is synchronized to the LD cycle (dark phase shown by shading) and in constant light (LL) the circadian rhythm free runs with a period of 23.97 hrs (Monkey Y). (**B**) Same animal, 4.8 months post-MPTP treatment, rest-wake activity is synchronized to the LD cycle but becomes arrhythmic in LL. Compared to pre-treatment, periodogram analyses (panels to right of actograms) confirm a quantitative decrease in amplitude during LD and the absence of rhythmicity during LL. The averaged 24 h locomotor activity profiles (**C–D**) illustrate the consolidated activity during the light phase in LD cycle both pre- and post-MPTP and during LL pre-MPTP but a complete loss of rhythmicity post-MPTP.

Under a daily 12 h/12 h light/dark cycle (LD), control (n = 4) experimental animals prior and post-MPTP treatment (n = 7) expressed a clear diurnal pattern of activity, although in the treated monkeys a number of behavioral timing parameters were less precise (**Fig. 1** **,** **2**) as has been previously reported [17], [39]. Compared to controls, MPTP animals showed a reduced activity during the light phase (91.5±0.6% vs 86.0±1.3%, P<0.01) and a decreased amplitude of the activity rhythm (χ^2^ = 7087±534 vs 4553±385, P<0.001). The activity onsets were also significantly less precise (**Fig. 3A**) and the rest-activity cycle was more fragmented and less stable (**Fig. 3E–F**). However, activity offsets and the phase angles of both activity onsets and offsets were not significantly affected after MPTP treatment (**Fig. 3B–D**).

**Figure 2:**
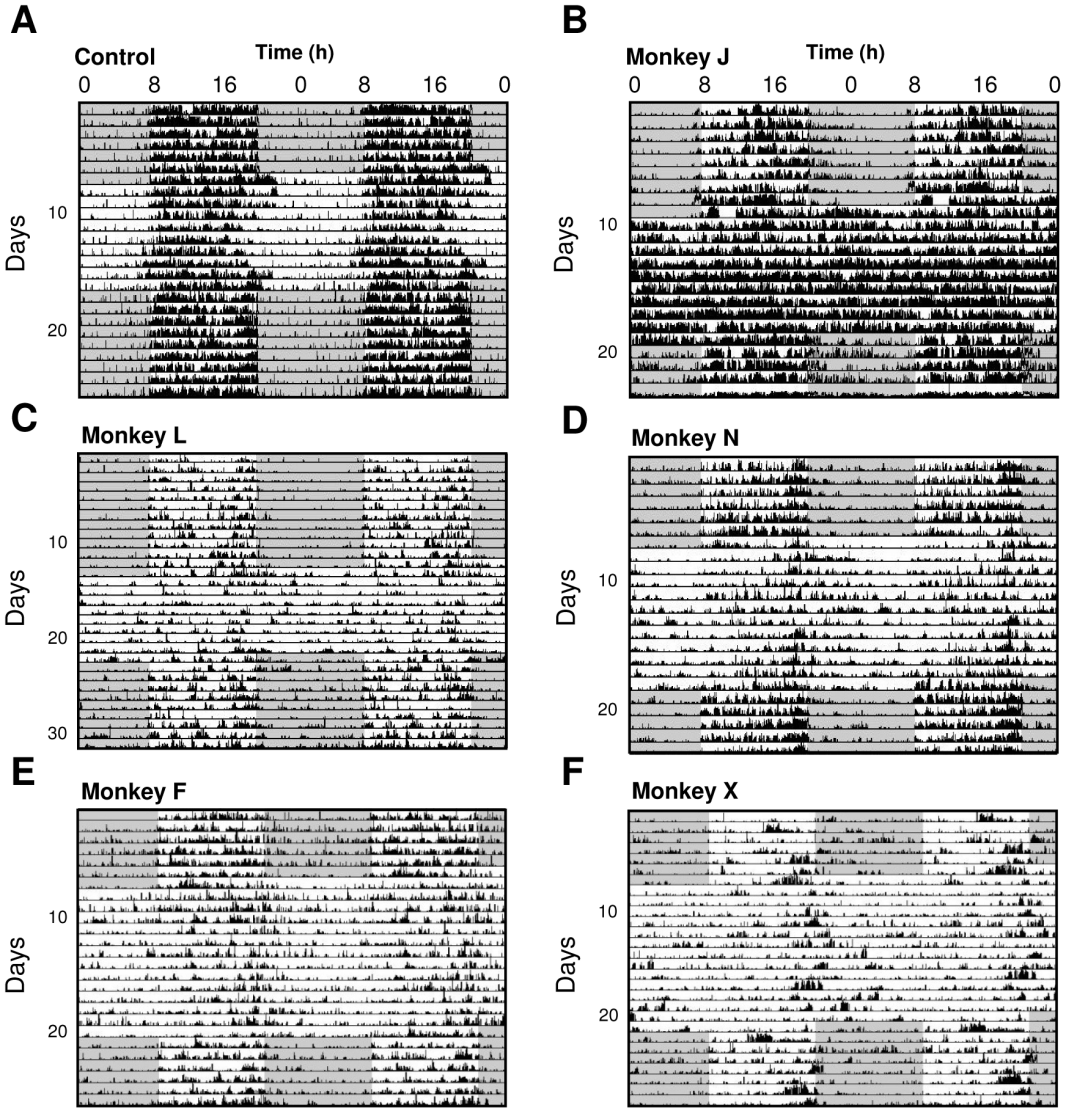
Double-plotted actograms illustrating alteration of circadian expression of locomotor activity in constant light (LL) in an untreated control and in 5 MPTP treated animals in addition to that shown in Figure 1. Note that while daily locomotor rest activity rhythms are observed for all animals during the LD cycle preceding and subsequent to LL. In MPTP-treated animals the circadian rhythm of locomotor activity was strongly altered (**D, E, F**) or abolished (**B, C**) in LL. **C** and **F** were highly symptomatic cases, **D** and **E** were mildly symptomatic and **B** was asymptomatic. The dark phase is shown by shaded areas and light phase by light areas.

**Figure 3:**
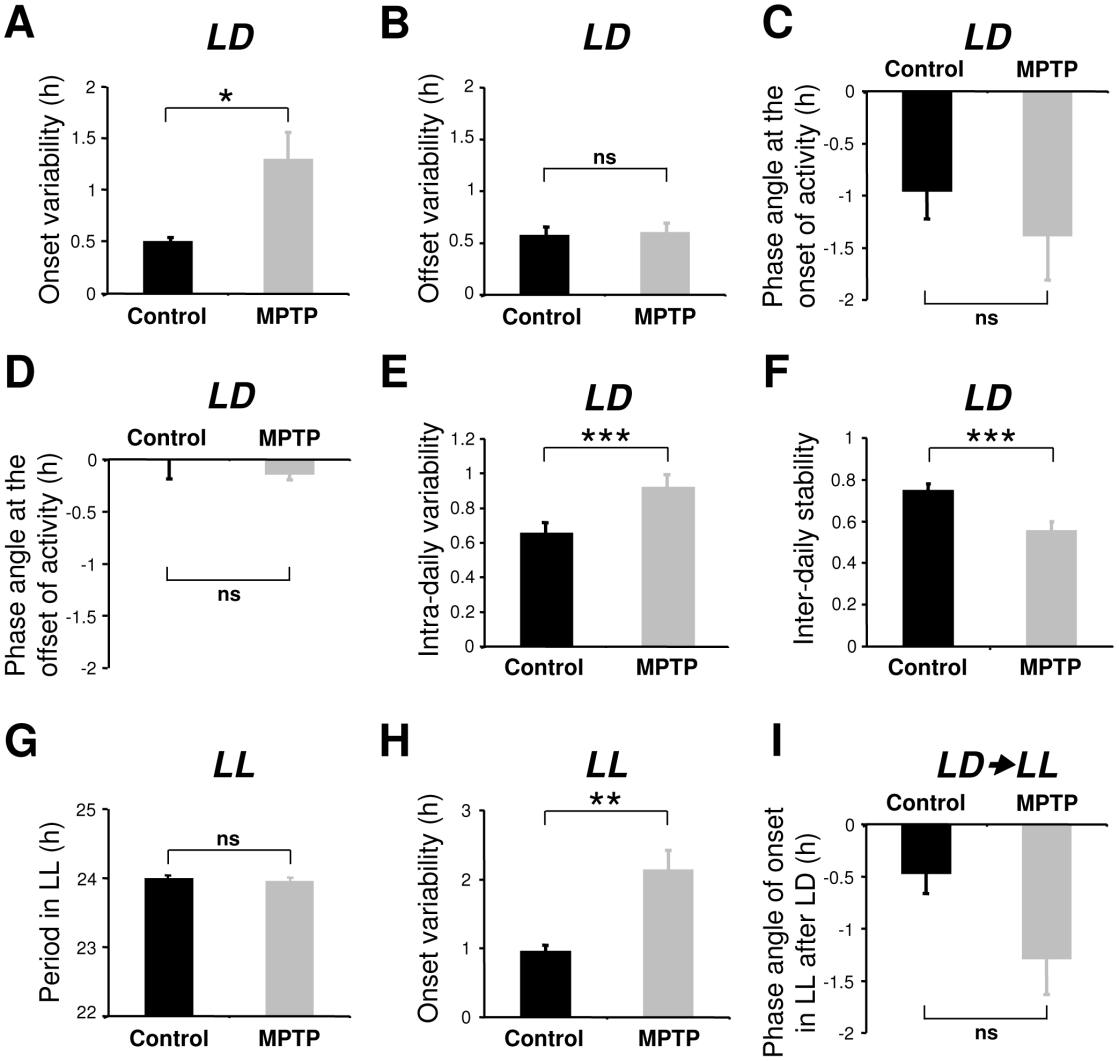
Variability of onsets, offsets, period, intra-daily variability, inter-daily stability and phase angle in control (n = 10) and MPTP-treated animals (n = 7). After MPTP treatment there was a significant increase of variability at the onset but not in the offset of locomotor activity in LD (**A, B**). Control and MPTP-treated animals show an approximately 1 hour phase delay in activity following lights on (**C**) and no phase angle difference in activity offset at lights off (**D**). The intra-daily variability increased (**E**) while the inter-daily stability decreased (**F**) after MPTP treatment. In LL the still rhythmic MPTP-treated animals showed an increased activity onsets variability (**H**), but with no significant difference in period (**G**). Upon release from LD to LL, MPTP animals had no significant difference in the phase angle of activity onset compared to controls (**I**) showing that the entrainment of activity rhythm under LD regime was not affected by MPTP. *P<0.05, **P<0.01, ***P<0.001.

When control animals were released into constant conditions (LL) they expressed a robust free running circadian rhythm with a mean period close to 24 h (23.99±0.04 h; **Fig. 1A**) with 89.4±0.7% of activity consolidated during the subjective day and a mean amplitude (χ^2^ periodogram) of 4,983±256. In contrast, 3 out of 7 MPTP-treated animals showed a complete loss of circadian rhythmicity (**Fig. 1B**, **2B–C**) with locomotor activity homogenously distributed throughout the 24 h period. The remaining treated animals showed extremely attenuated, low amplitude circadian rhythms (examples in **Fig. 2D–F**) coupled with highly imprecise activity onsets (**Fig. 3H**). Upon the transition from LD to LL conditions, the 4 rhythmic animals showed longer, but not significant, phase angle delays of activity onset compared to controls (**Fig. 3I**). When re-exposed to LD conditions, both rhythmic and arrhythmic animals regained rhythmic behavior similar to that expressed in LD before LL (**Fig. 1B** **,** **2**). Despite the obvious motor deficits subsequent to reduced DA levels, both control and MPTP-treated animals showed similar, insignificant relative decreases in the mean overall 24 h activity in LL compared to LD (control: 0.58±5.8%; MPTP: −16.70±6.24%) while one arrhythmic animal actually significantly increased its overall activity (+151±5.6%; P<0.001; **Fig. 2B**). This argues that the loss of circadian locomotor rhythmicity cannot be simply explained by motor impairments.

### Relation between circadian rhythmicity, clinical score and DA loss

**Figure 4:**
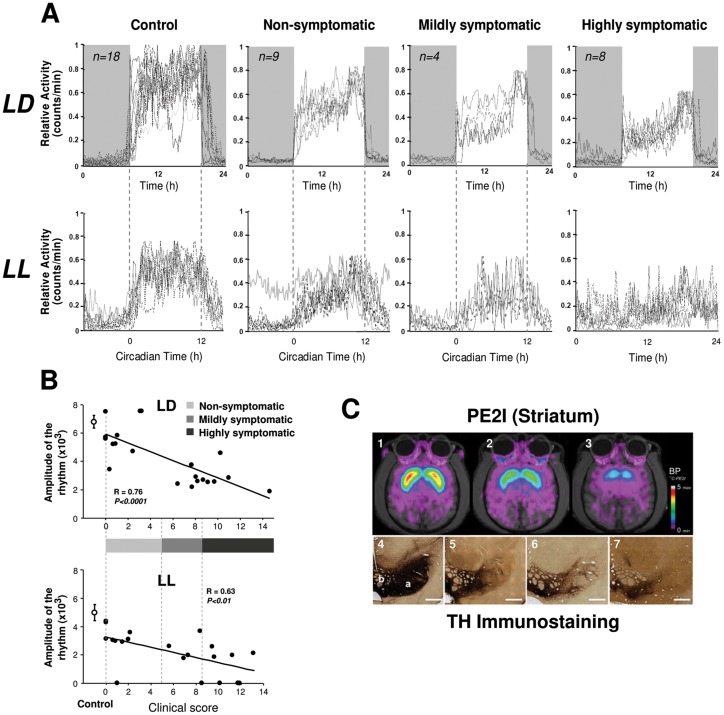
Averaged 24-activity profiles for all control and MPTP-treated monkeys with individual traces for all recordings (n = 39 assays) in LD (upper panel) and in LL (lower panel) conditions (A). Data for LD always correspond to the recording period immediately preceding LL. Control (or pre-treatment) animals show a robust daily and circadian rhythmicity with a consolidated activity during the light phase in LD and the subjective day in LL. In MPTP-treated animals, the daily rhythmicity of locomotor activity in the LD cycle is clearly evident but becomes progressively less robust in non-symptomatic, mildly symptomatic and highly symptomatic animals. When transferred to constant LL conditions, circadian rhythmicity deteriorates and highly symptomatic animals are arrhythmic with a continuous distribution of activity throughout the 24 h period. Note that the loss of rhythmicity is also recorded in one asymptomatic animal under LL (also shown in **Fig. 2B**). Relationship between the amplitude of the rhythm (χ^2^ periodogram) and clinical score in LD and LL (**B**). A complete loss of rhythmicity is mainly, but not exclusively observed for the highest clinical scores whereas some subjects with severe motor symptoms still maintained rhythmicity. Values are shown as mean ± SEM. MPTP treatment induced loss of dopamine neurons in the mesencephalon and dopaminergic depletion of the striatum (**C**). Examples of [^11^C]-PE2I scans of the basal ganglia before (**C_1_**) and after MPTP treatment showing 69% decrease in [^11^C]-PE2I binding potential in the striatum (**C_2_**) and 86% in a more extreme case (**C_3_**). The reduction of TH immunostaining in the mesencephalon of MPTP-treated monkeys (**C_5_**, 65%, **C_6_**, 72%, **C_7_**, >80%) compared to a control (**C_4_**). a: Substantia nigra (SN); b: Ventral tegmental area (VTA), Scale bar: 1000 µm.

It is well documented that in both PD patients and in MPTP-treated NHP there is a high degree of variability between individuals in the expression and severity of motor symptoms in relation to loss of DA neurons [39], [40]. We therefore examined the relationship between the clinical state of the animals and the amplitude of activity rhythms under LD and constant LL conditions. In an LD cycle, both control and MPTP animals maintained significant amplitudes of day-night rhythms with activity concentrated during the light phase regardless of the severity of parkinsonian motor symptoms (**Fig. 4A**). Under LL conditions, controls showed a robust circadian rhythmicity similar to that in LD (χ^2^ amplitude; 5252±500). Following MPTP treatment, animals in non-symptomatic and mildly symptomatic states showed distinct free-running rhythms but with significantly reduced amplitudes compared to controls (non-symptomatic, χ^2^ = 2890±447, P<0.001; mildly symptomatic, χ^2^ = 2831±361, P<0.001). One notable exception was a non-symptomatic animal assessed 7.3 months after MPTP treatment that displayed a robust day-night rhythmicity in LD conditions but a complete loss of circadian rhythm when exposed to LL (**Fig. 2B** **,** **4A**). Animals in a highly symptomatic state showed a marked dampening or a loss of rhythmicity in LL (**Fig. 4A**). In the MPTP animals that conserved a weak but significant circadian rhythm under constant conditions, there was no significant change in the period of the endogenous rhythm compared to controls (**Fig. 3E**).

To explore in greater detail the relationship between expression of circadian rhythms and severity of motor symptoms, we plotted the amplitude of the rhythm as a function of motor clinical score (**Fig. 4B**). In an LD cycle, there is an inverse correlation between χ^2^ amplitude and clinical score of the animals (P<0.0001) with the most severely motor impaired animals showing the lowest amplitudes of daily rhythms (**Fig. 4B**). In constant light, this correlation is weakened (P<0.01) such that the clinical score was a less accurate predictor of the loss of rhythm. For example, symptomatic animals with clinical PMRS scores between 5–14 could be either weakly rhythmic or totally arrhythmic. Arrhythmia was seen not only in symptomatic animals, but also in one completely non-symptomatic animal (**Fig. 4B**).

We verified the extent of dopaminergic degeneration in a longitudinal follow-up study using PET scans of [^11^C]-PE2I (a marker of DA transporters (DAT), [40], [41]) and post-mortem histological examination of TH and DAT protein levels in the substantia nigra, VTA and striatum. At the end of MPTP treatment, PET scans (n = 4) revealed an overall mean decrease of striatal [^11^C]-PE2I binding potential of 83±1.7% (**Fig. 4C**). The most affected structures were the putamen and the caudate nucleus (total reduction of 88±1.1%) compared to the nucleus accumbens that was less affected (74±1.6% reduction). The postmortem histological quantifications (n = 5) showed a comparable amount of reduction in the striatum for DAT (63±8.2%; PET-scan cases [n = 3]: 71±11.5%) and for TH immunoreactivity (55±6.7%; PET-scan cases [n = 3]: 57±11.8%). Reduction in mesencephalic structures (SN and VTA) averaged 40±4.6% for TH and 58±4.8% for DAT staining. Surprisingly, of the 4 animals that showed a final 82–90% reduction of [^11^C]-PE2I, 2 showed arrhythmia while 2 maintained a circadian rhythm in LL although the rest-activity cycle became less consolidated with a 50.3±0.33% reduction in amplitude. The clinical scores for these subjects ranged from 7 to 13. Furthermore, one monkey that had one of the lowest reduction in striatal DA (−46.6%, striatal DAT immunolabel), no clinical symptoms (score = 1) and a normal rhythm in an LD cycle was nevertheless arrhythmic in constant conditions.

### Endogenous circadian hormonal rhythms remain intact in MPTP-treated monkeys

**Figure 5:**
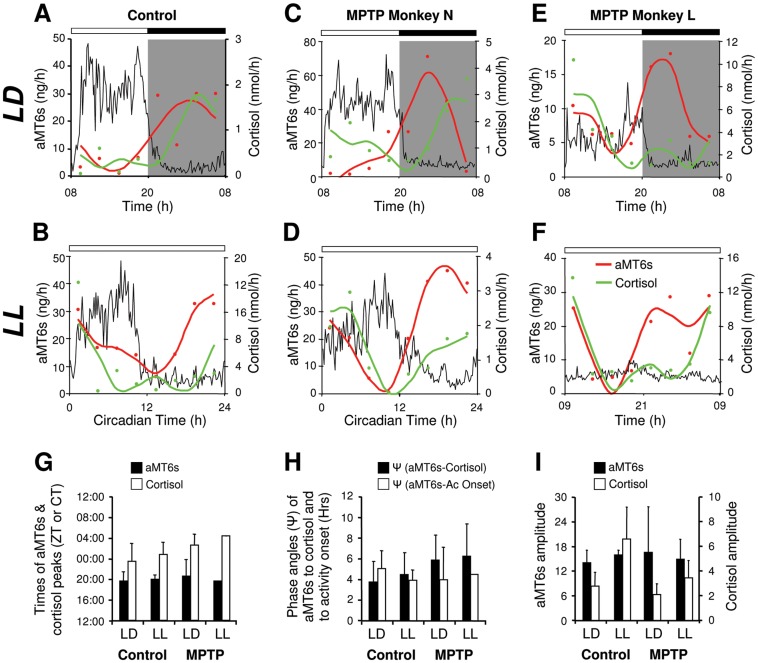
MPTP-treated monkeys maintain 24 h hormonal rhythms of melatonin and cortisol in both entrained (LD) and constant conditions (LL). Representative individual 24(dual harmonic regression fit) of urinary concentration of 6 sulphatoxymelatonin (red circles, line) and cortisol (green circles, line) compared to the mean relative activity profile (black line). Gray shading indicates the dark phase. In LD, controls and MPTP-treated animals show clear rhythms of locomotor activity, aMT6s and cortisol (**A, C, E**). In LL, control (**B**), rhythmic (**D**) and arrhythmic (**F**) MPTP animals also show a clear circadian rhythmicity of aMT6s and cortisol. The times of the aMT6s and cortisol peaks (**G**), the phase angles of aMT6s to both cortisol and activity rhythms (**H**) and the amplitudes of aMT6s and cortisol rhythms (**I**) were similar in LD and LL in both controls and MPTP-treated subjects (controls n = 4; MPTP n = 3).

The disruption or the loss of rhythmicity seen in MPTP-treated monkeys may suggest that the functional integrity of the SCN and/or the endogenous 24 h rhythm might no longer be intact [42], [43]. To explore this assumption, we assayed the rhythms of 6-sulphatoxymelatonin (aMT6s, the major metabolite of melatonin) and cortisol, two hormonal, gold-standard markers controlled by SCN output pathways independent from those controlling motor functions. Under LD entrainment conditions, both control and MPTP-treated animals show significant 24 h rhythmicity in aMT6s and cortisol (**Fig. 5**). In both controls and MPTP-treated animals the peak of aMT6s was observed as expected during the night and was in opposition of phase with the diurnal peak of activity, whereas the peak of cortisol occurred in early morning (**Fig. 5A, C, E, G**).

Under constant conditions, all control and MPTP-treated animals showed a significant circadian rhythmicity of both aMT6s and cortisol (**Fig. 5B, D, F**) with similar conservation of key rhythmic parameters. First, as in LD, the phase relationships of activity and hormonal rhythms were conserved and second, the peak of aMT6s occurred during the subjective night in opposition of phase with activity while high cortisol levels were found during the early subjective morning (**Fig. 5G–H**). In arrhythmic animals, the times of subjective day and night could not be determined due to the absence of an activity phase marker. Nevertheless, both aMT6s and cortisol excretions showed a significant circadian rhythm and retained their normal phase relationships (**Fig. 5F–H**). Furthermore, the amplitudes of aMT6s and cortisol were not affected by MPTP treatment (**Fig. 5I**). Together, these results are consistent with unimpaired output of SCN endogenous rhythmicity to the structures controlling these hormonal regulations.

### Masking effects of darkness on locomotor activity are unaltered following MPTP treatment

**Figure 6:**
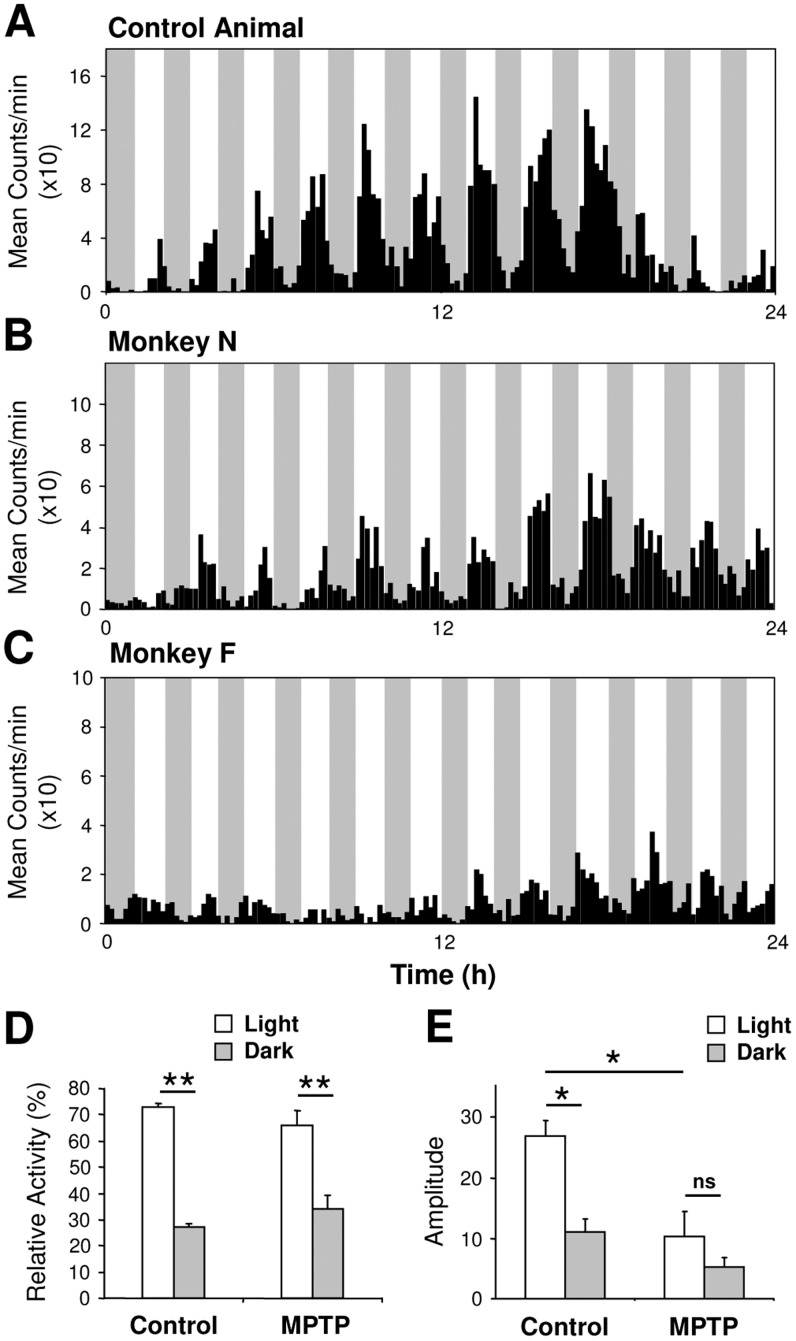
MPTP-treated monkeys show normal masking responses to darkness in a 1 h light–1 h dark regime. The control monkey (**A**) shows a clear 24 h rhythmicity coupled with a strong masking effect of darkness on locomotor activity. MPTP-treated monkeys show attenuated amplitude of the rhythm (**B**, mildly symptomatic, **C**, highly symptomatic) but nevertheless a strong masking effect of darkness proportionately similar to that of controls. (**D**) Mean relative total activity during light and dark phases. (**E**) Amplitude of the rhythm analysed separately for light and dark phases (controls n = 4; MPTP n = 5). *P<0.05, **P<0.001.

How can the maintenance of a daily rest-activity rhythm in an LD cycle in MPTP animals be explained given the absence of a circadian locomotor rhythm in constant conditions? In addition to entraining effects, light is known to exert an acute effect on locomotor activity known as masking [22], [23]. In nocturnal rodents, light strongly inhibits activity whereas in diurnal species, darkness is inhibitory [22], [23]. We explored the possibility that masking effects contribute to maintain an apparent synchronization of behavior in LD by assaying activity in a 1 h light/1 h dark cycle [22], [23], [44]. In this cyclic light regime, both control and MPTP monkeys showed strong masking effects of darkness on activity (**Fig. 6A–C**) with no significant differences in the relative amounts of locomotor activity expressed during the light phases (control = 73.1±1.6%, MPTP = 66.1±5.4%; **Fig. 6D**). Cosinor analysis showed a daily modulation of the average level of activity within both the light and dark phases in both control and MPTP animals with, as expected, higher amplitudes in the controls (**Fig. 6E**, P<0.05). The masking effect was observed for all treated monkeys regardless of the severity of motor impairments and despite a significant reduction in both the amount of overall activity (control = 4040.6±368.1 counts/24 h, MPTP = 2156.4±823.8 counts/24 h; P<0.001) and the amplitude of the rhythm (**Fig. 6E**).

### Absence of alterations in the SCN, hypothalamus and retina following MPTP treatment

**Figure 7:**
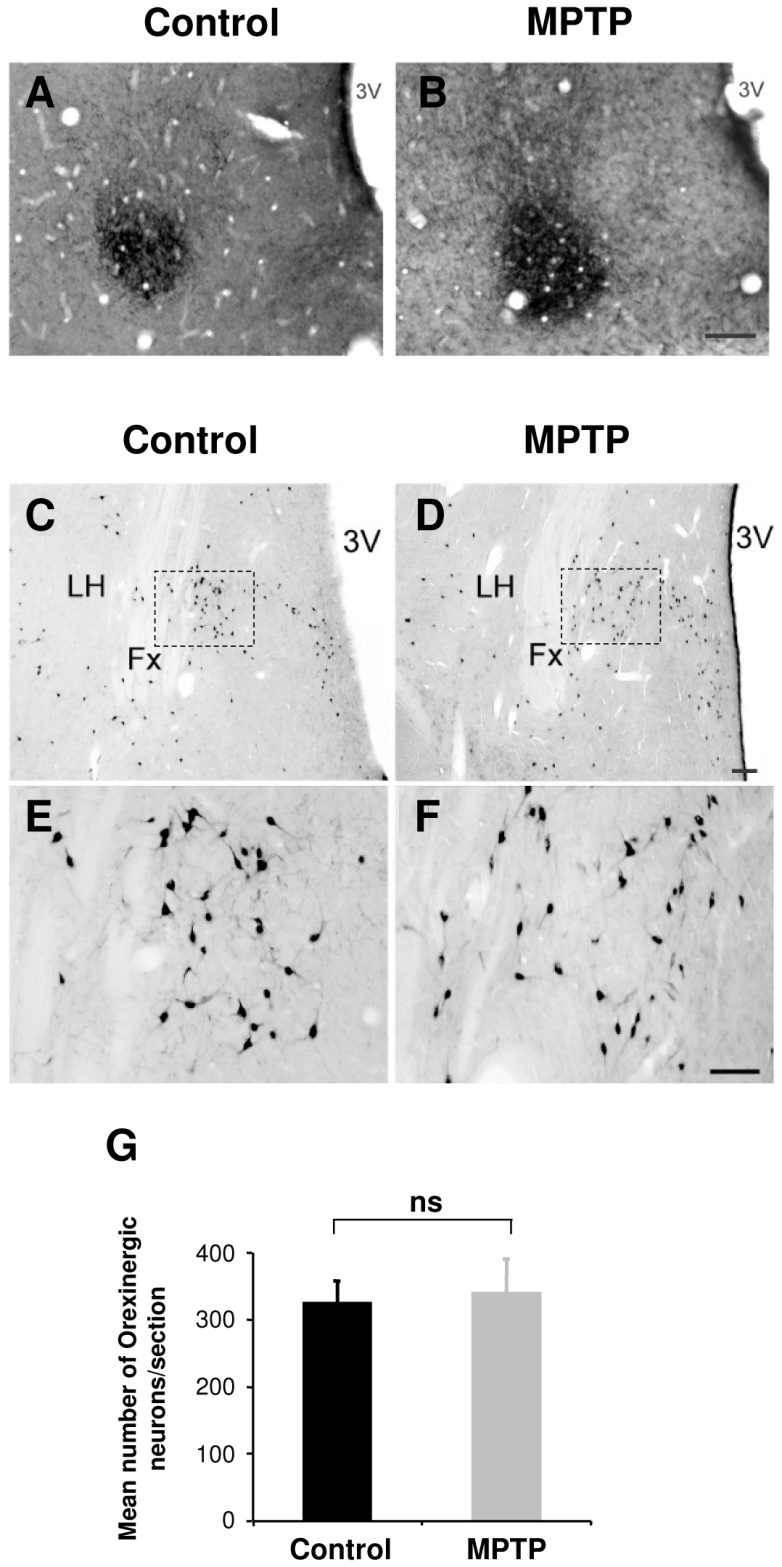
The orexinergic system in the lateral hypothalamic area and vasoactive intestinal peptide (VIP) containing neurons in the suprachiasmatic nucleus (SCN) are not affected after MPTP treatment. Example section in the hypothalamic SCN illustrating staining of VIP immunopositive neurons in a control (**A**) and an MPTP-treated animal (**B**). Scale bar = 100 µm. Example sections of orexin-A immunopositive neurons in the lateral hypothalamus of a control (**C, E**) and MPTP-treated animal (**D, F**). The regions indicated dashed square in **C** and **D** are shown at higher magnification in **E** and **F**. (**G**) There was no significant difference in the number of orexin expressing neurons between control (n = 1) and MPTP-treated animals (n = 3). All orexin-A expressing neurons in the hypothalamus were counted on 5–7 sections per animal (distance between sections 150–200 µm). Scale bar: **C, D** = 200 µm, **E, F** = 100 µm. LH = Lateral hypothalamus; Fx = Fornix; 3V = Third ventricle.

**Figure 8:**
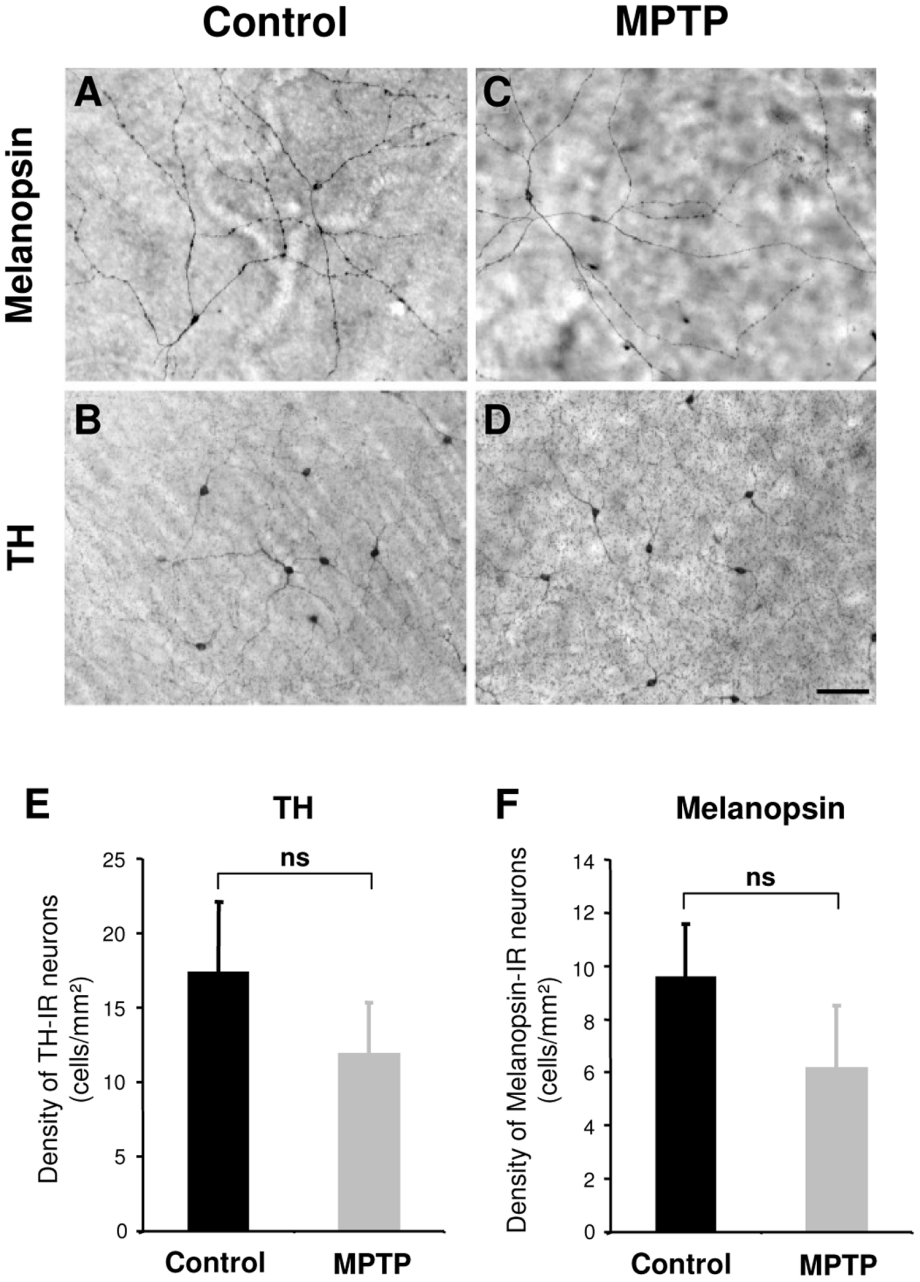
In the retina, the morphology of melanopsin and TH immunoreactive neurons appear normal after MPTP treatment. The photomicrographs are from representative flat-mounted retina immunostained for melanopsin (**A, C**) or TH (**B, D**) in control (**A, B**) and MPTP-treated animals (**C, D**). Scale bar: 100 µm. **E** and **F** illustrate histograms of the densities of TH and melanopsin immunoreactive neurons. (Controls n = 2; MPTP n = 3).

We examined in the available post-mortem materials the histological status of several retinal and hypothalamic systems. In the SCN, we found no qualitative differences between control and MPTP-treated animals in the intensity of immunohistochemical staining or the morphology of vasoactive intestinal peptide (VIP, **Fig. 7**) and vasopressin (not shown) containing neurons. The hypothalamic orexinergic system plays a crucial role in sleep-wake regulation and recent findings have suggested a reduction of orexinergic neurons and orexin levels in ventricular CSF in PD patients [45], [46]. However, in the MPTP-treated NHP, no differences were observed in the number, distribution or dendritic morphology of orexin-A immunoreactive neurons compared to controls (**Fig. 7C–G**). In the retina, we found no significant differences in the density or dendritic morphology of TH stained dopaminergic amacrine interneurons or melanopsin ipRGCs following MPTP treatment (**Fig. 8**).

## Discussion

The main finding of this study is that, in the absence of cyclic environmental day-night cues, DA depletion of the nigro-striatal pathway prevents the circadian system from effectively driving rhythmic rest-wake locomotor activity. This loss of rhythmicity was unexpected and could be interpreted as resulting from a defect of the SCN endogenous clock. However, the conservation of normal amplitude and phase relations of hormonal rhythms in constant conditions suggests that the SCN clock is not dysfunctional and that the observed deficit resides in the downstream output control of rhythmic locomotor functions.

Following MPTP treatment, increased motor impairment caused a decrease in the total daily amount of motor activity and consequently a reduction in the amplitude of activity rhythms. As in several previous studies of MPTP-treated monkeys in an LD cycle [16], [17], [39], rest-wake rhythms nevertheless remained phase synchronized with light and dark, with activity predominantly confined to the light phase. However, compared to control or pre-treatment state, MPTP monkeys showed less precision in their daily rhythm expressed as decreased amplitude and inter-daily stability and increased variability of activity onsets at lights-on and intra-daily variability. In contrast, the time and variability of activity offset at lights-off was as precise in MPTP-treated as in the control animals.

The environmental light-dark cycle exerts powerful synchronizing effects on the endogenous circadian clock of the SCN, thereby tightly controlling physiological and behavioral rhythms [43], [47], [48]. The rhythmic control of the circadian clock on output functions can thus only be assessed in constant conditions where these temporal influences of the light dark cycle are absent. Mouse models with striatal DA lesions show various alterations of rest activity rhythms in both LD and DD, but a loss of circadian rhythmicity in constant conditions has not been observed [49]. Previous studies in PD patients and NHP models of the disease have not investigated alteration of locomotor and physiological rhythms under these conditions [16]–[21], [39], [50]. In LL, normal diurnal monkeys show robust circadian rhythms (present study, [51], [52]) as do nocturnal rodents in constant darkness [49]. We found that MPTP-treated monkeys exposed to constant light displayed highly disturbed circadian rest-activity rhythms, ranging from a poorly consolidated daytime activity of extremely low amplitude to complete loss of the circadian rhythm. Animals with greater motor deficits generally showed a reduced amplitude of rhythmicity in both LD and LL conditions compared to non- or mildly symptomatic states. However, a high clinical score or loss of DA function as measured by [^11^C]-PE2I or DAT and TH in the SN were not reliable predictors of a complete loss of circadian locomotor rhythmicity. It has long been recognized that MPTP treated monkeys show large inter-individual variability in the severity of motor impairments in relation to DA loss, and in their vulnerability to neurotoxic treatment, possibly associated with differences in compensatory mechanisms [53], [54]. In our study, one animal that showed no clinical symptoms for several months following the end of MPTP treatment and a normal daily rhythm in LD conditions, nevertheless completely lacked a circadian rhythm in LL associated with a significant increase of continuous 24 h activity. The loss of rhythmicity seen in our MPTP-treated animals cannot therefore be considered as an epiphenomenon of MPTP-induced hypokinesia.

A loss of circadian expression of rhythmicity is a severe state that typically requires interference with, or impairment of, the core molecular clock mechanism in the SCN. Arrhythmia is typically observed following lesion of the SCN [42], [43] or invalidation of core clock genes (*Per*, *Cry*, *Bmal*, *Clock*, [43]). Despite the absence of a functional clock in these cases, a daily rhythm of behavior can often be observed in the presence of a light-dark cycle, whereas upon release into constant conditions rhythmicity is abolished. Although in our NHP model we were unable to probe clock gene expression directly, a deficiency of the clock mechanism does not appear to be responsible for the loss of the activity rhythm. Indeed, hormonal rhythms of melatonin and cortisol are preserved with normal phase relationships in LD and LL, confirming that an endogenous rhythm is present in the SCN and is capable of driving these output pathways. These results are also consistent with previous studies that report little or no alteration of several hormonal rhythms in PD patients and MPTP-treated NHP [18]–[21]. Similar observations have made in Alzheimer patients, suggesting that the loss of circadian control of activity/rest cycles is not a consequence of a global circadian disruption but rather involves a dysfunction of SCN pathways that specifically control rest activity behavior while outputs to other systems remains unaffected [55].

Under LD conditions, light not only synchronizes rest-activity behavior through the circadian system, but also exerts an independent acute effect on locomotor activity known as masking [22], [23]. Light strongly inhibits activity of nocturnal rodents whereas in diurnal species, darkness is inhibitory [22], [23]. In MPTP monkeys, an unaltered photic light detection is consistent with the conservation of melanopsin ipRGCs and the strong masking effects of darkness in parkinsonian monkeys, even in the highly symptomatic, arrhythmic cases. The robust inhibitory effect of darkness is further exemplified by the abrupt offset of activity caused by lights off in the LD cycle in all animals. These combined effects of stimulatory and inhibitory influences of light plausibly explain the maintenance of daily rhythms of activity in MPTP-treated NHP under an LD cycle.

Since the SCN oscillator controlling hormonal rhythms appears intact, the loss of circadian sleep-wake rhythmicity in the MPTP monkey likely lies downstream from the SCN, in the control of locomotor output behaviors. DA is a necessary component for normal motor behavior and is required to drive the expression of locomotor output activity [56]. The primary deficit following MPTP treatment is the depletion of DA in the striatum. Virtually all aspects of dopaminergic transmission (DA, TH, DA receptors, DAT transporter and the main DA metabolites DOPAC & HVA) show daily rhythms of expression in the striatum [7]–[9]. Several of these components are also light and clock dependent since the diurnal rhythm is abolished or damped by lesion of the SCN [10], [11]. Light also exerts an acute activating effect on DA in the substantia nigra and the striatum [57]. Finally, *in vivo* and *in vitro* studies further suggest DA mediated regulation of clock genes in striatal neurons [8], [12], [13]. The absence of DA rhythmicity in the rat striatum alters, but does not prevent a daily rhythm of activity whereas the circadian rhythm is abolished in constant darkness [8], [13]. Per2 levels and cycling in the striatum but not in the SCN are also affected, suggesting a local role of DA in the regulation of both clock genes and behavioral output [13]. Similar results have been described in DA deficient flies that show a decreased entrainment in LD but normal masking responses to light [58]. These studies in rodents and flies, along with the present findings in NHP, support the idea that not merely DA per se, but rhythmic dopaminergic function is critical for the normal expression of rhythmic clock gene and control of rest-wake behavior. The importance of this circadian pattern of DA function is in accordance with observations that continuous infusion of DA medication during waking hours yields superior outcomes with fewer side effects compared to discontinuous or continuous 24 h round-the-clock administration [59].

The regulation of sleep-wake behavior is governed by two distinct processes, a sleep-dependent mechanism (sleep homeostat) and the circadian sleep regulatory mechanism [14], [15]. The timing and quality of sleep can be modulated by independent or concomitant manipulation of these two systems suggesting that a time-dependent sleep disorder may result from a dysfunction in one or both processes. It would therefore have been interesting to also monitor EEG in our monkeys, however, this was difficult to achieve in the framework of the present protocol. There is increasing evidence that in addition to the primary dopaminergic insult, neuronal alterations and lewy body pathologies occur in many non-dopaminergic sleep/wake control centers in PD. These include adenosine neurotransmission [66], noradrenerigic neurons in the Locus coeruleus [67]–[69], serotonergic neurons in the raphe [67], cholinergic neurons in the peduncular pontine nucleus and basal forebrain [70] and orexinergic neurons in the lateral hypothalamus [45], suggesting the involvement of homeostatic process dysfunction as a contributing factor in the sleep/wake cycle abnormalities. In MPTP-treated NHP, however, a similar template of dysfunction has not been documented and many of these features; lewy bodies pathology [66], [71], loss of serotoninergic neurons in the raphe [72] and cholinergic neurons in the PPN and the basal forebrain [73] appear to be lacking. Our study additionally shows no occurrence of neuronal loss of orexinergic neurons in the lateral hypothalamus.

**Figure 9:**
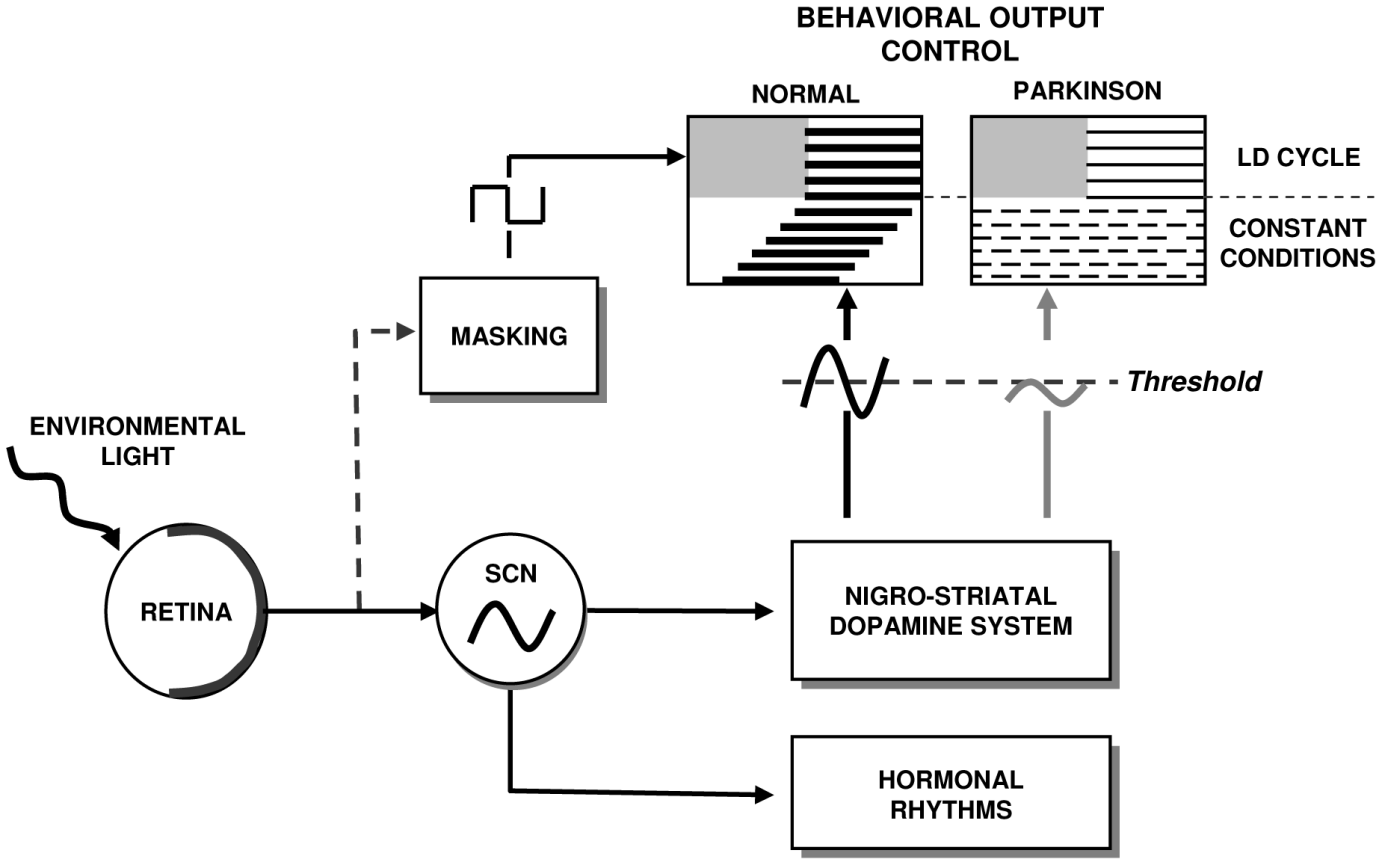
Model of the consequences of dopamine depletion in the nigrostriatal system. In healthy subjects the LD cycle synchronizes daily output rhythms (activity, hormones) and fine tunes these rhythms through acute masking effects. In constant conditions, rhythmic outputs free-run under the control of the circadian clock. In the case of dopamine depletion in the nigrostriatal system, the excitatory and inhibitory effects of environmental light and dark are sufficient to sustain expression of a daily locomotor rhythm. In constant conditions, the endogenous clock is intact and drives rhythmic hormonal outputs. In contrast, rhythmic locomotor behavior is abolished if the rhythmic dopamine function driven by the SCN falls below a hypothetical threshold.

Our results suggest that in the neurotoxic MPTP treated NHP, the retinal input and core clock mechanism of the circadian timing system remain essentially intact but are unable to efficiently drive rhythmic locomotor activity. In normal animals, both the clock-dependent and the activating acute effects of light control the rhythmic expression of DA transmission that in turn drives rhythmic striatal gene expression and behavior. Following DA depletion, the combined stimulatory effects of light and inhibitory effect of darkness on the remaining dopaminergic and other systems [22] may be sufficient to sustain a daily rest-activity pattern in an LD cycle. However, in constant conditions these acute effects are lacking. We propose that the inability of the circadian system to drive rhythmic locomotor activity in the absence of environmental timing cues can be attributed to a downstream effect involving disconnection of the central circadian clock from its output target networks controlling locomotor function (see model in **Fig. 9**). As DA levels decrease following neurotoxicity, one can hypothesize that rhythmic dopaminergic neurotransmission falls below a hypothetical threshold necessary for overt locomotor rhythms. An alternative but not exclusive hypothesis is that DA may be required to synchronize the phase relationships of individual oscillatory neurons and to generate coherent systems level output of the striatal system, in which case the function of DA as a coupling factor in the striatum would be analogous to that of VIP in the SCN [60].

The disconnection of circadian control of the dopaminergic system implies that exposure to a sufficiently luminous day-night cycle is critical for the maintenance of rhythmic dopaminergic function. This may be highly critical for elderly PD patients, particularly in health care facilities, as previous studies have shown that in elderly patients, increasing the ambient light levels during the day improves rest-activity rhythms, sleep quality, alertness and well-being [61]. In northern latitudes where the circadian system is seasonally confronted with continuous day or night conditions it is tempting to speculate that rest-activity cycle and sleep disturbances in PD may be exacerbated during the summer or winter. Indeed, several epidemiological studies report increased occurrences of PD in northern compared to southern latitudes [62]–[64]. Our results are of clinical importance and stress that sleep/wake disturbances associated with DA loss may be more severely affected than previously thought, in particular in sub-optimal lighting conditions. In the light of beneficial effects of long-term light therapy on motor and non-motor symptoms of PD [65], our study further emphasizes that the circadian component of PD related symptoms must be considered in therapeutic approaches along with classical DA treatments.
